# The Association Between Obesity and Low Back Pain and Disability Is Affected by Mood Disorders: A Population-Based, Cross-Sectional Study of Men

**DOI:** 10.1097/MD.0000000000003367

**Published:** 2016-04-18

**Authors:** Louisa Chou, Sharmayne R.E. Brady, Donna M. Urquhart, Andrew J. Teichtahl, Flavia M. Cicuttini, Julie A. Pasco, Sharon L. Brennan-Olsen, Anita E. Wluka

**Affiliations:** From the Department of Epidemiology and Preventative Medicine (LC, SREB, DMU, AJT, FMC, JAP, AEW), School of Public Health and Preventative Medicine, Monash University; Baker IDI Heart and Diabetes Institute (AJT), Melbourne; School of Medicine (JAP, SLB-O), Deakin University, Geelong; North-West Academic Centre (JAP, SLB-O), The University of Melbourne; and Australian Institute of Musculoskeletal Sciences (SLB-O), Melbourne, Victoria, Australia.

## Abstract

Low back pain (LBP) and obesity are major public health problems; however, the relationship between body composition and low back pain in men is unknown. This study aims to examine the association between body composition and LBP and disability in a population-based sample of men, as well as the factors that may affect this relationship.

Nine hundred seventy-eight male participants from the Geelong Osteoporosis Study were invited to participate in a follow-up study in 2006. Participants completed questionnaires on sociodemographics and health status. Low back pain was determined using the validated Chronic Back Pain Grade Questionnaire and the presence of an emotional disorder was assessed using the Hospital Anxiety Depression Scale. Body composition was measured using dual energy x-ray absorptiometry.

Of the 820 respondents (84% response rate), 124 (15%) had high-intensity low back pain and/or disability (back pain). Low back pain was associated with higher body mass index (28.7 ± 0.4 vs 27.3 ± 0.2 kg/m^2^, *P* = 0.02) and waist–hip ratio (0.97 ± 0.006 vs 0.96 ± 0.006, *P* = 0.04), with increased tendency toward having a higher fat mass index (8.0 vs 7.6 kg/m^2^, *P* = 0.08), but not fat-free mass index (*P* = 0.68). The associations between back pain and measures of obesity were stronger in those with an emotional disorder, particularly for waist–hip ratio (*P* = 0.05 for interaction) and fat mass index (*P* = 0.06 for interaction).

In a population-based sample of men, high-intensity LBP and/or disability were associated with increased levels of obesity, particularly in those with an emotional disorder. This provides evidence to support a biopsychosocial interaction between emotional disorders and obesity with low back pain.

## INTRODUCTION

Low back pain is a major public health problem and was identified as the leading cause of disability worldwide in the Global Burden of Disease Study.^1,2^ Approximately 80% of adults experience at least 1 episode of back pain during their lifetime.^3^ Despite the magnitude of the problem, the structural origin of most episodes of back pain is unknown, with poor correlation between symptoms and structural abnormalities.^4^ Hence back pain is usually considered “non-specific.” In order to address this problem, research has focused on identifying modifiable risk factors for back pain.

Potential risk factors for back pain include older age, female sex, lower educational attainment, increased physical work demands, and emotional disorders.^5^ Obesity has also been linked with low back pain,^6^ although a previous systematic review concluded obesity was only a weak risk factor.^7,8^ It is estimated that approximately one-third of the world's adult population is overweight, as defined by body mass index (BMI).^9^ Obesity may have both biomechanical and meta-inflammatory effects on the spine.^10^ However, obesity measured by BMI is a crude measure of adiposity, as it fails to differentiate fat from fat-free mass. Moreover, body composition is markedly different between males and females,^11,12^ with higher fat mass in females compared with males. This may account for the results of a meta-analysis that concluded that the association between being overweight or obese and low back pain is stronger in women compared with men.^13^ While this gender disparity in the relationship between obesity and back pain may be related to differences in pain perception and hormonal influences,^14^ differences in the composition of fat and fat-free mass between males and females may also play a role. Fat mass has been shown to be associated with back pain intensity and disability in a cohort of predominantly female adults,^15^ but this has yet to be comprehensively examined in males. This is a particularly pertinent question since the burden of low back pain in men is estimated to be higher than in women, as measured by disability-adjusted life years.^16^

The aim of this study was to examine the association between body composition and low back pain and disability in a population-based sample of men, as well as the factors that may affect this relationship.

## METHODS

### Study Population

The GOS is a population-based Australian study, designed to investigate the epidemiology of osteoporosis among adults. During the baseline study conducted from 2001 to 2006, an age-stratified sample of 1540 men was randomly recruited from the Barwon Statistical Division using the Australian electoral roll as a sampling frame. This study evaluated adult male participants (n = 978) aged ≥20 years who participated in the 5-year follow-up of the Geelong Osteoporosis Study (GOS) from 2006 to 2010.^17^ Reasons for loss to follow-up: 141 had died before the follow-up, 41 had left the region, 16 were unable to provide informed consent, 139 were not contactable, and 225 declined to participate.^17^ Thus, the remaining 978 participants (81%) of the potential study population attended a clinical assessment that included measures of body composition, as well as the completion of questionnaires designed to assess demographics, health status, and back pain. The Human Research Ethics Committees of Barwon Health and Monash University approved this study. All participants provided informed consent.

### Data Collection

#### Main Outcome: Low Back Pain and Disability

Low back pain intensity and disability in the past 6 months were evaluated using the Chronic Pain Grade Questionnaire, a validated tool used to grade the severity of chronic pain and disability in population-based studies in primary care settings.^18,19^ It includes 7 questions from which a pain intensity score (0–100) and disability points score (0–6) are calculated. Participants are initially classified into 1 of 5 groups based on the Chronic Pain Grade Classification, as the tool was intended; no pain and disability (pain intensity score = 0 and disability points = 0), low intensity pain and low disability (pain intensity score <50 and disability points <3), high intensity pain and low disability (pain intensity score ≥50 and disability points <3), high disability that is moderately limiting (disability points 3 or 4, regardless of pain intensity), and high disability that is severely limiting (disability points 5 or 6, regardless of pain intensity). Participants were further categorized into 2 groups; no or low back pain intensity and disability (no pain and disability or low intensity pain and low disability) or high back pain intensity and/or disability (high intensity pain and low disability, or high disability that is moderately limiting or high disability that is severely limiting).

#### Demographics and General Health

Self-reported information was obtained by questionnaires. Education was determined using the question, “What is your highest completed level of education?” with 6 possible answers (no school, primary school, some secondary school, completed secondary school, postsecondary qualification, tertiary qualification). Secondary school in Australia is education provided for students typically aged between 13 and 18 years of age, therefore for analyses, participants were categorized as being either completers or noncompleters of secondary school. Mobility was assessed by using the question, “How would you best describe your activity now?” with 7 possible answers, with descriptors, available (very active, active, sedentary, limited, inactive, chair or bedridden, and bedfast). For analyses, the nominal data was were then categorized into 2 groups to differentiate those who were physically mobile (very active or active) and those who had poor mobility (sedentary, limited, inactive, chair or bedridden, and bedfast), as previously applied.^20^ “Clinically significant anxiety and/or depressive symptomatology was determined by use of the validated Hospital Anxiety and Depression Scale (HADS). The self-report HADS tool measures 7 items each for anxiety (HADS-A) and depression (HADS-D), using a 4-point ordinal scale for each to define symptoms from none to most severe (0–3, respectively). Using a cut-point of ≥8 of the total HADS score to indicate high symptomatology, a binary variable was created, as previously employed.^21^ We have not differentiated anxiety from depression, and have utilized, the combined scale (anxiety and/or depression score) to indicate the presence of an emotional disorder.^21,22^”

#### Measures of Body Composition

Weight was measured to the nearest 0.1 kg and height was measured to the nearest 0.001 m using a stadiometer (with shoes and bulky clothing removed). From these data, BMI [weight (kg)/height (m^2^)] was calculated. Body composition was measured using dual-energy x-ray absorptiometry (DXA; GE Lunar Prodigy, GE Lunar Corp, Madison, WI). Whole body measures of body fat (fat mass = FM) and lean tissue mass (fat-free mass = FFM) were determined. Based on these data, fat-mass index (FMI) was calculated as FMI = fat-mass/height^2^ and fat-free mass index (FFMI) was calculated as FFMI = fat-free mass/height^2^ (where fat-free mass = lean tissue mass + bone mineral content). The ratio between FM and FFM (fat mass/fat-free mass) was also calculated.

#### Statistical Analysis

Independent-sample *t* tests and *χ*^2^ tests were used to detect differences in age, emotional disorder, education, mobility, and body composition between participants with no or low pain and disability and those with high pain intensity and/or high disability. Binary logistic regression was used to examine associations between demographic factors and high-intensity low back pain and/or disability and estimated marginal means were used to assess the relationships between obesity measures and body composition in participants with high-intensity pain and disability compared to those with no or low pain and disability. Multivariate analyses included adjustments for age, emotional disorder, education, mobility, and BMI. To examine the multivariate associations between body composition (FM or FFM) and back pain, adjustment was made for the alternate body composition measure; for example, when fat mass was the exposure of interest, multivariate analyses were also adjusted for fat-free mass. Interactions between risk factors for low back pain and measures of obesity, including measures of body composition, were examined. A *P* value of less than 0.05 was regarded as statistically significant. All analyses were conducted using SPSS Statistics 22.0 (SPSS Institute, Chicago, IL).

## RESULTS

**FIGURE 1:**
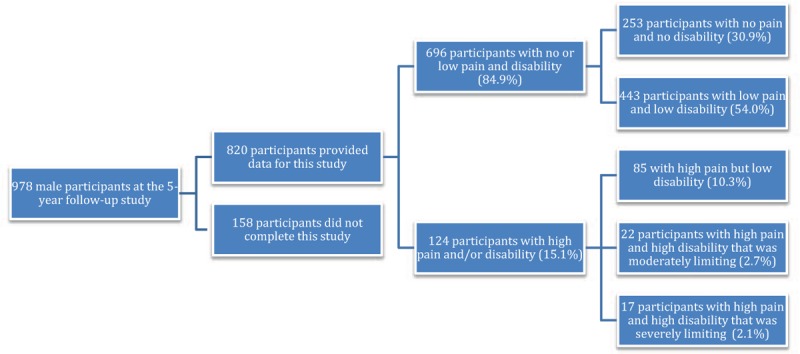
Flow diagram showing the number of participants based on low back pain intensity and/or disability.

Eight hundred and twenty (83.8%) of the potential 978 male participants provided data for this study. No differences were detected between the 158 men who did not complete this study and those who did in terms of age, emotional disorder, education, mobility, and measures of obesity and body composition (*P* > 0.07 for all, results not shown). Of the participants, 696 (84.9%) had no or low pain and disability, with 253 (30.9%) having no pain and no disability and 443 (54.0%) having low pain and low disability. There were 124 (15.1%) participants with high pain and/or disability, with 85 (10.3%) having high pain but low disability, 22 (2.7%) having high disability that was moderately limiting, and 17 (2.1%) having high disability that was severely limiting (see Figure 1).

The characteristics of men with versus without high-intensity back pain and/or high disability were compared (Table 1). Participants with high disability and/or high-intensity pain were older, more likely to have an emotional disorder, less likely to have completed secondary school and more likely to have poor mobility than those with no or low back pain and disability (*P* < 0.002 for all). They were also heavier (*P* = 0.01) and had a higher BMI (*P* = 0.001) and waist–hip ratio (*P* = 0.001). In terms of body composition, participants with high-intensity pain and/or disability had higher fat mass and fat mass index (*P* < 0.001 for both) and fat mass/fat-free mass ratio (0.74 vs 0.66, *P* ≤ 0.001). There were no differences detected in fat-free mass and fat-free mass index between those with versus without high pain and/or disability (*P* > 0.34 for both).

**TABLE 1:**
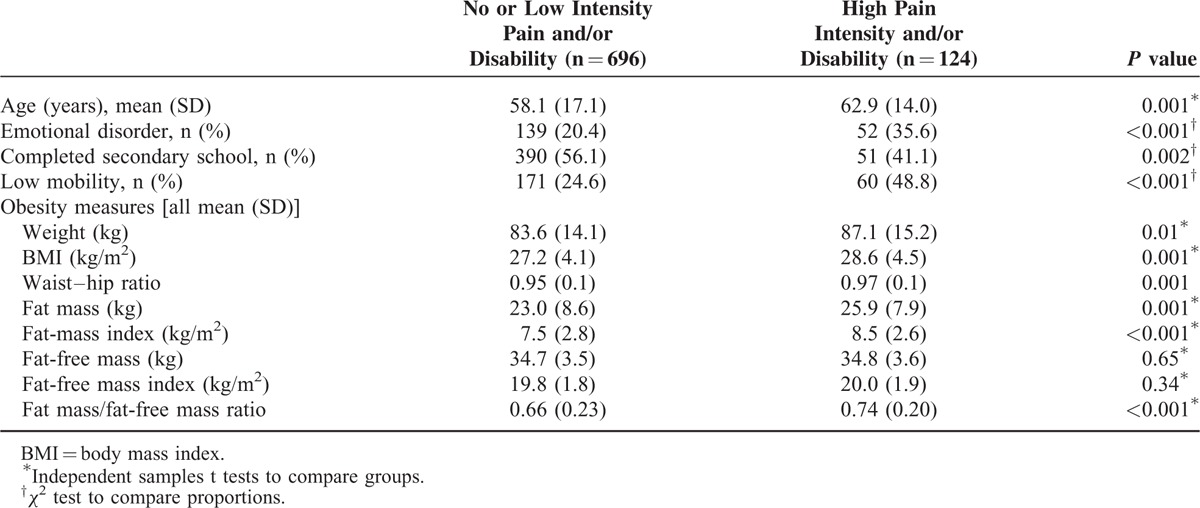
Comparison of Participants With No or Low Pain and Disability Versus Those With High Pain Intensity and/or Disability

The associations between demographic factors and high-intensity back pain and/or high disability were examined, adjusted for potential confounding variables, using estimated marginal means (Table 2). The presence of an emotional disorder and poor mobility was associated with having high-intensity back pain and/or disability (*P* < 0.001 for both). There was also a trend toward having high-intensity pain and/or disability with increasing age (*P* = 0.06). Those who had not completed secondary school associated had reduced odds of high pain intensity and/or disability, although this was not statistically significant (*P* = 0.06).

**TABLE 2:**
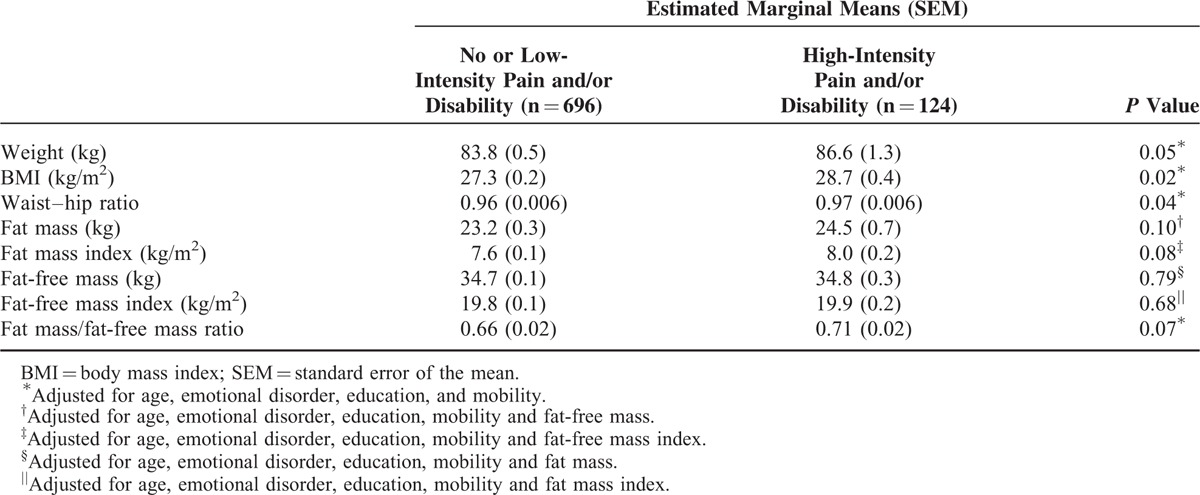
Association Between Obesity and Body Composition and Low Back Pain and/or Disability

Measures of obesity and body composition were adjusted for age, emotional disorder, education and mobility, and compared in men with versus without high-intensity pain and/or disability, as shown in Table 2. Men with high-intensity pain and/or disability had a higher BMI (28.7 vs 27.3 kg/m^2^, *P* = 0.02) and waist–hip ratio (0.97 vs 0.96, *P* = 0.04) and tended to be significantly heavier (86.6 vs 83.8 kg, *P* = 0.05) after adjustment. There was a tendency for men with high-intensity pain and/or disability to have a higher fat mass (24.5 vs 23.2 kg, *P* = 0.10) and fat mass index (8.0 vs 7.6 kg/m^2^, *P* = 0.08) than men with no or low pain and disability. Fat-free mass and fat-free mass index were not significantly different between those with versus without high pain and/or disability (*P* > 0.68 for both).

We examined for interactions between risk factors for low back pain and measures of obesity and low back pain. The association between measures of obesity and low back pain tended to be stronger in those with an emotional disorder, than those without (Table 3). There was a trend toward statistically significant interactions between the presence of an emotional disorder with waist–hip ratio (*P* = 0.05) and fat mass index (*P* = 0.06). Hence, of the participants who had an emotional disorder, the association between higher waist–hip ratio or fat mass index with low back pain was stronger than in those without an emotional disorder. Participants who had an emotional disorder were similar in terms of mobility, education, and obesity measures (data not shown, *P* > 0.18), compared to those without an emotional disorder. There was no evidence of statistically significant interactions between measures of obesity and age (*P* > 0.13 for all), education (*P* > 0.12 for all), or mobility (*P* > 0.16 for all) and the presence of high-intensity back pain and/or disability.

**TABLE 3:**
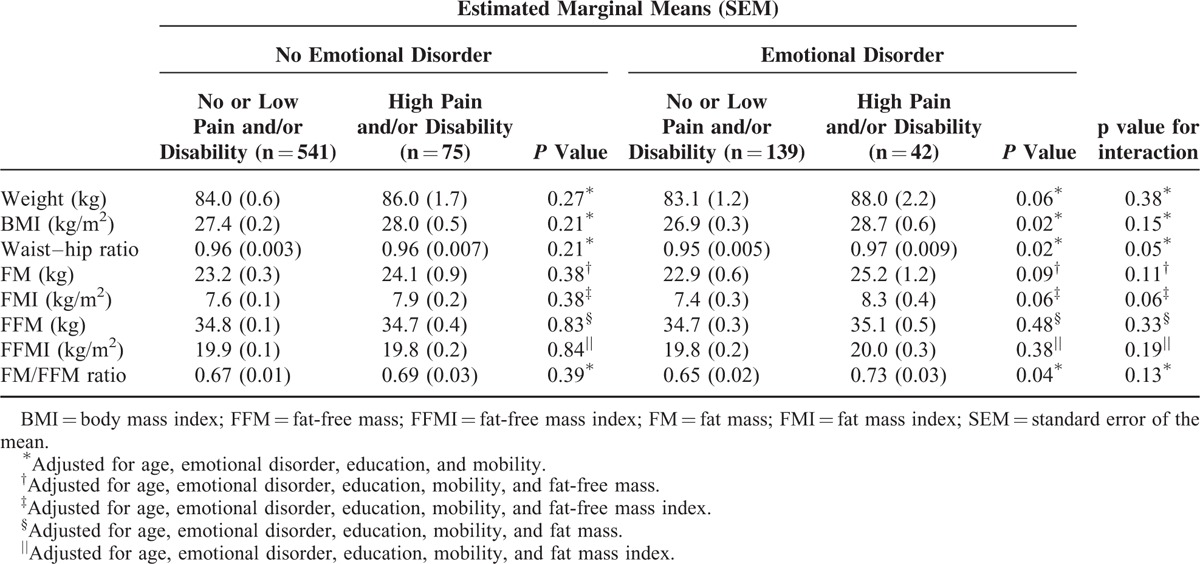
The Presence of an Emotional Disorder Affects The Association Between Obesity and Body Composition Measures and High-Intensity Back Pain and/or Disability

## DISCUSSION

In a population-based cohort of men, higher BMI and waist–hip ratio were associated with high-intensity low back pain and/or high disability, after adjusting for confounders such as age, emotional disorder, education, and mobility. Other measures of increased adiposity, such as weight, fat mass index, and fat mass/fat-free mass also tended to be associated with high-intensity back pain and/or disability. However, there was no evidence that measures of lean tissue mass, assessed using fat-free mass, and fat-free mass index were similarly associated with back pain. Furthermore, the association between measures of metabolic obesity (waist–hip ratio and fat mass index) and back pain was stronger in those who had an emotional disorder than those who did not have an emotional disorder.

These findings raise the possibility that although biomechanical factors related to spinal loading are associated with back pain, a systemic metabolic process associated with excess adipose tissue may also play a role in back pain and disability. Obesity has previously been demonstrated to be a risk factor for back pain.^7^ Some prior studies used only weight, BMI, and waist–hip ratio to measure obesity:^6,7,13^ these measures do not provide information on body composition. It is important to consider the role of body composition, as emerging evidence suggests that fat and muscle mass have different roles in the pathogenesis of musculoskeletal pain. For instance, fat mass but not muscle mass was associated with incident foot pain^22^ and musculoskeletal pain at multiple sites has also been associated with fat mass in women but not men.^23^ Similarly, we showed in a smaller population of predominantly women, that fat mass, but not lean mass was associated with higher levels of back pain and disability.^15^ Thus, the current study is the first to evaluate the association between obesity measures including body composition and low back pain in a large population-based sample of men. We have demonstrated that in men increased BMI and waist–hip ratio are associated with high levels of back pain and/or disability. It is possible that the previous studies did not adequately account for potential confounders, such as education and mental health, or it may be that these relationships differ between men and women. It is biologically plausible that a gender disparity in the pathophysiology of back pain would exist. This might be related to the differences in fat distribution, with men typically having an android distribution of fat, which is concentrated around the abdomen and upper body, compared with the gynoid distribution that is common in women where fat is increased around the hips and thighs.^24^ An android fat distribution may result in a stronger impact of biomechanical factors related to spinal loading causing higher compressive force on lumbar spine structures on the development of low back pain.

When we examined the association between measures of obesity and back pain with regards to mental health, we found that the associations tended to be stronger in those with an emotional disorder. We considered whether this might simply be because those who are obese may be more likely to be depressed and/or anxious and thus more likely to experience back pain. However, we found no differences in degree of obesity between participants with an emotional disorder versus those without. Thus, we need to consider how the combination of obesity and the presence of an emotional disorder may interact and influence the likelihood of having back pain. Obesity, depression and anxiety are now recognized as being inflammatory states.^25–27^ Adipose tissue, particularly when deposited in an androgenic distribution around the torso rather than evenly distributed throughout the body, is considered a metabolically active organ.^28^ This metabolically active adipose tissue produces hormones, such as leptin, oestrogen, and resistin and proinflammatory cytokines, such as tumor necrosis factor-alpha and interleukin-6.^29–31^ These adipokines have been independently related to the accelerated onset of depression,^32^ as well as osteoarthritic changes in both the spine^33^ and the knee.^34,35^ Higher levels of proinflammatory cytokines have also been shown to have a relationship with the progression to chronic pain^36^ and elevated levels of circulating C-reactive protein, a marker of systemic inflammation, has been identified as a risk factor for depression.^37^ Furthermore, the dysregulation of inflammatory cytokine production in depression and anxiety can also potentiate pain pathways.^38,39^ People with an emotional disorder may be sensitized to experience pain,^40^ which may be further exacerbated by meta-inflammation due to increased adiposity, as measured by increased waist–hip ratio and fat mass index. Therefore, the combination of having an emotional disorder and being obese supports a biopsychosocial role of adipose tissue in the pathophysiology of low back pain. This raises the possibility of future strategies for back pain management to specifically target fat loss in those with an emotional disorder.

There are a number of limitations to our study. First, the cross-sectional design of our study precluded the examination of any temporal relationships between fat mass and low back pain: longitudinal studies are needed. Although the Chronic Pain Grade Questionnaire has been shown to be a valid and reliable measure of pain intensity and disability in population-based studies, and has been used in many studies in this context of chronicity,^18,15^ it does not specify how many days of pain and/or disability participants had over the past 6 months. Also, the ability of the HADS to discriminate between anxiety and depression is controversial; therefore, the combined HADS score was used rather than the HADS subscales.^41^ Moreover, data were missing for 16.2% of eligible participants; however, there were no significant differences in age, emotional disorders, education, and measures of obesity between those who completed this study and those who did not. While we did not have a measurement of physical activity which is known confounder for low back pain,^42^ we did take into account a measure of mobility as an indicator of how active the participants were. Also, we did not analyze other variables such as the participants’ occupation or number or type of medical comorbidities in our analysis; however, we were able to adjust for a broad range of potential confounders such as age, emotional disorders, education, and mobility.

This study had a number of considerable strengths. This study evaluated a large population-based sample of men, whereas previous studies were either small or had a predominance of women in their study.^15,43^ Furthermore, GOS participants were randomly recruited from the Australian electoral roll and the study region has been shown to be representative of the broader Australian population.^17^ We used a validated questionnaire to measure back pain intensity and disability^18,19^ whereas some previous studies have not^43^ and we used a number of unique measures of adiposity.

This study demonstrated that obesity was associated with high levels of low back pain and disability in a population-based cohort of men. In particular, for men with a concomitant emotional disorder, back pain was more likely to be associated with increased adiposity (i.e. waist–hip ratio and fat mass index). These findings highlight the importance of obesity as a modifiable risk factor for back pain and suggest a biopsychosocial interaction between and obesity with low back pain. Although these findings will need to be confirmed in longitudinal studies, they have important implications for prevention and treatment of back pain and disability in men.

## References

[1] Buchbinder R Blyth FM March LM Placing the global burden of low back pain in context. *Best Pract Res Clin Rheumatol* 2013; 27:575–589. 24315140 10.1016/j.berh.2013.10.007

[2] Vos T Flaxman AD Naghavi M Years lived with disability (YLDs) for 1160 sequelae of 289 diseases and injuries 1990-2010: a systematic analysis for the Global Burden of Disease Study 2010. *Lancet* 2012; 380:2163–2196. 23245607 10.1016/S0140-6736(12)61729-2 PMC6350784

[3] Rubin DI Epidemiology and risk factors for spine pain. *Neurol Clin* 2007; 25:353–371. 17445733 10.1016/j.ncl.2007.01.004

[4] Jensen MC Brant-Zawadzki MN Obuchowski N Magnetic resonance imaging of the lumbar spine in people without back pain. *N Engl J Med* 1994; 331:69–73. 8208267 10.1056/NEJM199407143310201

[5] Hoy D Brooks P Blyth F The epidemiology of low back pain. *Best Pract Res Clin Rheumatol* 2010; 24:769–781. 21665125 10.1016/j.berh.2010.10.002

[6] Heuch I Heuch I Hagen K Body mass index as a risk factor for developing chronic low back pain: a follow-up in the Nord-Trondelag Health Study. *Spine* 2013; 38:133–139. 22718225 10.1097/BRS.0b013e3182647af2

[7] Leboeuf-Yde C Body weight and low back pain. A systematic literature review of 56 journal articles reporting on 65 epidemiologic studies. *Spine* 2000; 25:226–237. 10685488 10.1097/00007632-200001150-00015

[8] Dario AB Ferreira ML Refshauge KM The relationship between obesity, low back pain, and lumbar disc degeneration when genetics and the environment are considered: a systematic review of twin studies. *Spine J* 2015; 15:1106–1117. 25661432 10.1016/j.spinee.2015.02.001

[9] Lehnert T Sonntag D Konnopka A Economic costs of overweight and obesity. *Best Pract Res Clin Endocrinol Metab* 2013; 27:105–115. 23731873 10.1016/j.beem.2013.01.002

[10] Shiri R Solovieva S Husgafvel-Pursiainen K The role of obesity and physical activity in non-specific and radiating low back pain: the Young Finns study. *Semin Arthritis Rheumatism* 2013; 42:640–650. 23270761 10.1016/j.semarthrit.2012.09.002

[11] Camhi SM Bray GA Bouchard C The relationship of waist circumference and BMI to visceral, subcutaneous, and total body fat: sex and race differences. *Obesity (Silver Spring)* 2011; 19:402–408. 20948514 10.1038/oby.2010.248 PMC3960785

[12] Pasco JA Nicholson GC Brennan SL Prevalence of obesity and the relationship between the body mass index and body fat: cross-sectional, population-based data. *PLoS One* 2012; 7:e29580. 22253741 10.1371/journal.pone.0029580 PMC3258232

[13] Shiri R Karppinen J Leino-Arjas P The association between obesity and low back pain: a meta-analysis. *Am J Epidemiol* 2010; 171:135–154. 20007994 10.1093/aje/kwp356

[14] Craft RM Mogil JS Aloisi AM Sex differences in pain and analgesia: the role of gonadal hormones. *Eur J Pain* 2004; 8:397–411. 15324772 10.1016/j.ejpain.2004.01.003

[15] Urquhart DM Berry P Wluka AE 2011 Young Investigator Award Winner. Increased fat mass is associated with high levels of low back pain intensity and disability. *Spine* 2011; 36:1320–1325. 21270692 10.1097/BRS.0b013e3181f9fb66

[16] Hoy D March L Brooks P The global burden of low back pain: estimates from the Global Burden of Disease 2010 study. *Ann Rheum Dis* 2014; 73:968–974. 24665116 10.1136/annrheumdis-2013-204428

[17] Pasco JA Nicholson GC Kotowicz MA Cohort profile: Geelong osteoporosis study. *Int J Epidemiol Dec* 2012; 41:1565–1575. 10.1093/ije/dyr148 23283714

[18] Smith BH Penny KI Purves AM The chronic pain grade questionnaire: validation and reliability in postal research. *Pain* 1997; 71:141–147. 9211475 10.1016/s0304-3959(97)03347-2

[19] Von Korff M Ormel J Keefe FJ Grading the severity of chronic pain. *Pain* 1992; 50:133–149. 1408309 10.1016/0304-3959(92)90154-4

[20] Hayley AC Williams LJ Kennedy GA Excessive daytime sleepiness and body composition: a population-based study of adults. *PLoS One* 2014; 9:e112238. 25383556 10.1371/journal.pone.0112238 PMC4226485

[21] Snaith RP The hospital anxiety and depression scale. *Health Quality Life Outcomes* 2003; 1:29. 10.1186/1477-7525-1-29 PMC183845 12914662

[22] Butterworth PA Urquhart DM Cicuttini FM Fat mass is a predictor of incident foot pain. *Obesity* 2013; 21:E495–E499. 23512967 10.1002/oby.20393

[23] Yoo JJ Cho NH Lim SH Relationships between body mass index, fat mass, muscle mass, and musculoskeletal pain in community residents. *Arthritis Rheumatol* 2014; 66:3511–3520. 25185757 10.1002/art.38861

[24] Marcus MA Wang J Pi-Sunyer FX Effects of ethnicity, gender, obesity, and age on central fat distribution: comparison of dual x-ray absorptiometry measurements in white, black, and Puerto Rican adults. *Am J Hum Biol* 1998; 10:361–369. 10.1002/(SICI)1520-6300(1998)10:3<361::AID-AJHB11>3.0.CO;2-6 28561402

[25] Vogelzangs N Beekman ATF de Jonge P Anxiety disorders and inflammation in a large adult cohort. *Transl Psychiatry* 2013; 3:e249. 23612048 10.1038/tp.2013.27 PMC3641413

[26] Liukkonen T Räsänen P Jokelainen J The association between anxiety and C-reactive protein (CRP) levels: results from the Northern Finland 1966 Birth Cohort Study. *Eur Psychiatry* 2011; 26:363–369. 21570260 10.1016/j.eurpsy.2011.02.001

[27] O’Donovan A Hughes BM Slavich GM Clinical anxiety, cortisol and interleukin-6: evidence for specificity in emotion–biology relationships. *Brain Behavior Immunity* 2010; 24:1074–1077. 10.1016/j.bbi.2010.03.003 PMC4361085 20227485

[28] Liu P Ma F Lou H The utility of fat mass index vs. body mass index and percentage of body fat in the screening of metabolic syndrome. *BMC Public Health* 2013; 13:629. 23819808 10.1186/1471-2458-13-629 PMC3703297

[29] Ronti T Lupattelli G Mannarino E The endocrine function of adipose tissue: an update. *Clin Endocrinol* 2006; 64:355–365. 10.1111/j.1365-2265.2006.02474.x 16584505

[30] Galic S Oakhill JS Steinberg GR Adipose tissue as an endocrine organ. *Mol Cell Endocrinol* 2010; 316:129–139. 19723556 10.1016/j.mce.2009.08.018

[31] Zhuo Q Yang W Chen J Metabolic syndrome meets osteoarthritis. *Nat Rev Rheumatol* 2012; 8:729–737. 22907293 10.1038/nrrheum.2012.135

[32] Pasco JA Jacka FN Williams LJ Leptin in depressed women: cross-sectional and longitudinal data from an epidemiologic study. *J Affect Disord* 2008; 107:221–225. 17727958 10.1016/j.jad.2007.07.024

[33] Igarashi A Kikuchi S Konno S Correlation between inflammatory cytokines released from the lumbar facet joint tissue and symptoms in degenerative lumbar spinal disorders. *J Orthop Sci* 2007; 12:154–160. 17393271 10.1007/s00776-006-1105-y

[34] Berry PA Jones SW Cicuttini FM Temporal relationship between serum adipokines, biomarkers of bone and cartilage turnover, and cartilage volume loss in a population with clinical knee osteoarthritis. *Arthritis Rheum* 2011; 63:700–707. 21305502 10.1002/art.30182

[35] Stannus OP Cao Y Antony B Cross-sectional and longitudinal associations between circulating leptin and knee cartilage thickness in older adults. *Ann Rheum Dis* 2015; 74:82–88. 24078677 10.1136/annrheumdis-2013-203308

[36] Dina OA Green PG Levine JD Role of interleukin-6 in chronic muscle hyperalgesic priming. *Neuroscience* 2008; 152:521–525. 18280048 10.1016/j.neuroscience.2008.01.006 PMC2336107

[37] Pasco JA Nicholson GC Williams LJ Association of high sensitivity C-reactive protein with de novo major depression. *Br J Psychiatry* 2010; 197:372–377. 21037214 10.1192/bjp.bp.109.076430

[38] Berk M Williams LJ Jacka FN So depression is an inflammatory disease, but where does the inflammation come from? *BMC Med* 2013; 11:200. 24228900 10.1186/1741-7015-11-200 PMC3846682

[39] Hayley S Poulter MO Merali Z The pathogenesis of clinical depression: stressor- and cytokine-induced alterations of neuroplasticity. *Neuroscience* 2005; 135:659–678. 16154288 10.1016/j.neuroscience.2005.03.051

[40] Gerrits MM Vogelzangs N van Oppen P Impact of pain on the course of depressive and anxiety disorders. *Pain* 2012; 153:429–436. 22154919 10.1016/j.pain.2011.11.001

[41] Cosco TD Doyle F Ward M Latent structure of the hospital anxiety and depression scale: a 10-year systematic review. *J Psychosomatic Res* 2012; 72:180–184. 10.1016/j.jpsychores.2011.06.008 22325696

[42] Lin CW McAuley JH Macedo L Relationship between physical activity and disability in low back pain: a systematic review and meta-analysis. *Pain* 2011; 152:607–613. 21251757 10.1016/j.pain.2010.11.034

[43] Toda Y Segal N Toda T Lean body mass and body fat distribution in participants with chronic low back pain. *Arch Internal Med* 2000; 160:3265–3269. 11088088 10.1001/archinte.160.21.3265

